# Brief online acceptance and commitment therapy for adults with type 1 diabetes: a pilot study

**DOI:** 10.3389/fcdhc.2024.1378946

**Published:** 2024-04-24

**Authors:** Cristina Stefanescu, Alin Laurentiu Tatu, Aurel Nechita, Claudia I. Iacob, Eugen Secara, Silvia Nicolescu, Gabriela Alexandra Huiu

**Affiliations:** ^1^ Faculty of Medicine and Pharmacy, “Dunărea de Jos” University, Galați, Romania; ^2^ Dermatology Department, “Sfanta Cuvioasa Parascheva” Hospital of Infectious Diseases, Galati, Romania; ^3^ Multidisciplinary Integrated Center of Dermatological Interface Research MIC-DIR, “Dunărea de Jos” University, Galati, Romania; ^4^ Department of Applied Psychology and Psychotherapy, Faculty of Psychology and Educational Sciences, University of Bucharest, Bucharest, Romania; ^5^ Faculty of Psychology and Educational Sciences, Babes-Bolyai University, Cluj Napoca, Romania

**Keywords:** type 1 diabetes, stress, online act, online acceptance and commitment therapy, psychological flexibility

## Abstract

Recognizing the pivotal role of psychosocial factors in triggering and maintaining Type 1 Diabetes Mellitus (T1DM), the integration of psychoeducational and psychotherapeutic interventions is associated with comprehensive management of the disease. This study aimed to evaluate the impact of a four-week online individual Acceptance and Commitment Therapy (ACT) intervention in mitigating diabetes-associated stress, fostering diabetes acceptance, enhancing the patient-doctor relationship, and promoting psychological flexibility in adults diagnosed with T1DM. Employing a single-arm trial design with mixed methodology, thirteen participants (Mage = 39.04, SDage =13.74) engaged in the intervention and completed self-report measures before and after the intervention. Quantitative analysis with the Wilcoxon signed-rank test indicated a statistically significant difference in diabetes-associated stress, diabetes acceptance, and psychological flexibility pre- and post-intervention. Notably, stress levels and psychological inflexibility diminished, while psychological flexibility and diabetes acceptance improved. However, the patient-doctor relationship did not exhibit a significant change. Furthermore, narrative feedback obtained from participants reflected overall satisfaction with the intervention. These preliminary findings contribute to the expanding body of literature supporting the affirmative influence of ACT interventions on individuals with T1DM.

## 1 Introduction

Diabetes is a chronic and intricate condition that requires a multifaceted approach for risk mitigation alongside glycemic control. Continuous diabetes self-management education and support are imperative for averting short-term complications and reducing the likelihood of enduring adversities (1). Type 1 diabetes mellitus (T1DM) is a persistent condition associated with substantial global mortality rates and considerable financial implications. The etiology of T1DM involves pancreatic cell demise, leading to insufficient insulin production (2). Psychosocial determinants, such as stress, mental health issues, cognitive patterns, and patient-doctor relationships, wield considerable influence over the management and progression of this disorder (3). Psychological interventions, notably acceptance and commitment therapy (ACT), have garnered significant research attention for optimal T1DM control. Consequently, we sought to evaluate the efficacy of ACT in this trial involving T1DM patients.

Individuals with T1DM encounter a spectrum of challenges encompassing disease-related variables, mental health disorders, cognitive patterns, and interactions with healthcare providers. Elements influencing the diabetes diagnosis, including disease severity and the support extended by the social network to patients (4–6), may either facilitate or hinder adjustment to the diagnosis. In addition, fundamental psychological aspects such as cognitive processes and psychological traits may play a significant role in adaptive capacities (7).

Hypoglycemia has notable cognitive and emotional effects on individuals with T1DM. Specifically, during periods of acute hypoglycemia, individuals experience heightened self-awareness and distraction when engaged in mentally demanding activities (1). This implies that the cognitive resources available for completing tasks are reduced, potentially impacting cognitive performance. Also, hypoglycemia was associated with anxiety, which is likely to have broad implications, influencing various aspects of the individual’s life, including social interactions, personal activities, and work-related tasks. A comprehensive review that included 68 research and data from 2,128,029 people showed that the incidence of anxiety disorders in diabetic patients was 28%, and individuals with anxiety disorders had a 19% increased risk of diabetes (2). Diabetes patients were 41% more likely to have anxiety problems. A comprehensive study revealed significant incidence of depression and diabetes comorbidity (3). Depression is more than three times more common in individuals with T1DM (12% vs. 3.2%) and nearly twice as common in those with type 2 diabetes (19.1% vs. 10.7%). Women with and without diabetes had a greater rate of depression than males (4).

A positive patient-doctor relationship (PDR) was linked to improved treatment adherence, patient satisfaction, and overall prognosis (5). Patients’ perceptions of doctor empathy, general trust, and faith in the medical doctor’s kindness predicted their appraisal of the patient-doctor interaction (6). Research on irritable bowel syndrome indicates that a PDR fostering mutual understanding and aiding patients in comprehending their symptoms positively impacts disease management in a psychologically adaptive manner (7). Thus, psychological flexibility (i.e. the person’s capacity to modify their behavior in response to shifting circumstances while concurrently maintaining alignment with their values and objectives (8) may be an indirect strategy for improving PDR. The characteristics of PDR share similarities with the helping alliance observed in psychotherapy, including elevated levels of trust, helpfulness, empathic understanding, and interpersonal openness (9). Although the relationship between ACT and PDR was not directly investigated as far as we know, there is evidence that ACT improves interpersonal relationships and anger management in male students (10), interpersonal skills in adolescents (11), and can optimize interpersonal problems in adults (12).

Given the significant role played by psychological variables in the onset and persistence of T1DM, psychoeducational and psychotherapeutic interventions have a crucial role in disease management. Individuals with diabetes are encouraged to consider cognitive-behavioral therapies (CBT), as these interventions have demonstrated efficacy in enhancing adherence to medical regimens (13). CBT emerges as an effective modality for patients with diabetes, leading to notable reductions in hemoglobin A1c levels, fasting blood sugar, diastolic blood pressure, as well as improvements in symptoms of depression and anxiety, along with enhanced sleep quality (13, 14).

Furthermore, research indicates the effectiveness of Acceptance and Commitment Therapy (ACT) as a third-wave cognitive-behavioral intervention in managing diabetes patients. Notably, three randomized controlled trials employing group-based ACT interventions revealed that participants in the ACT groups exhibited greater enhancements in diabetes self-management, treatment adherence, and the adoption of effective coping strategies when compared to control groups (15) treatment adherence (15) and effective coping strategies (16) compared to control groups. Based on other studies, ACT was beneficial in lowering depression in patients with type 2 diabetes mellitus (17, 18). ACT was also useful in managing other chronic conditions, such as irritable bowel syndrome (19), chronic pain (20), epilepsy (21), substance use disorders (22).

ACT is a transdiagnostic intervention that can be administered as individual modules focusing on specific processes associated with psychological flexibility (23). The “Hexaflex” captures six connected processes in psychological flexibility: contact with the present moment, values, committed action, self as context, defusion, and experiential acceptance. These processes improve the well-being and health of people with diabetes by allowing them to be attentive to and accept their internal experiences as they are and seek to live a meaningful life based on their particular values (24). Psychological flexibility may be a significant factor to consider when selecting and customizing intervention options for diabetes self-management, acceptance, and coping patterns. For example, if psychological inflexibility is related to the level of diabetes distress and how patients deal with their diabetes self-management tasks, a multidisciplinary team can address inflexibility and/or consider how to reduce the burden of the diabetes regimen (25).

ACT is a developing field of research, as shown in a systematic review of 17 papers with diverse study designs (26). In this review, six trials included follow-up, and all employed pre- and post-intervention assessments. Twenty general and diabetes-specific psychological outcomes were evaluated. The behavioral outcomes were mostly related to diabetic self-management, whereas the physical outcomes were primarily concerned with glycated hemoglobin (HbA1c) levels, a marker of glycemic control, during the previous three months. Seven papers examined ACT mechanisms. The study cohort comprised 827 individuals of diverse nationalities, with a substantial proportion being of Iranian origin. Fourteen studies targeted adults, while three were focused on pediatric populations. Group interventions (n = 13) were the most frequently studied. The number of sessions ranged from one to 15 and the overall duration of the intervention ranged from one to 90 days. The trials included all ACT components (26).

What has not yet been explored in the current literature is the relationship between medical doctors and patients and how ACT can be associated with changes in the PDR. Although this study reports preliminary results, we expect to see positive changes in PDR. Moreover, the majority of previous studies explored the impact of ACT intervention in group settings, while our approach involves the individual delivery of the intervention. Very few studies have compared the differences between individual versus group ACT. However, initial evidence suggests that there is no difference between the two formats in terms of treatment outcomes in teenagers with chronic pain (27). For the purpose of this study, we chose the individual format, due to major differences in participants’ schedule and availability. Additionally, this approach allows for the treatment to be highly personalized to the individual’s specific needs, experiences, and challenges related to managing T1DM. Lastly, the study aimed to assess the impact of a brief online individual ACT intervention in managing diabetes-associated stress, diabetes acceptance, PDR, and increasing psychological flexibility in adults with T1DM. The decision to use a brief intervention model aligns with the goal of providing timely and focused support to individuals managing T1DM. Brief interventions, even single-session ACT protocols, have been shown to be effective for a range of psychological issues, offering practical tools and strategies that can be quickly learned and applied (28). We selected the online format to increase attainment and accommodate their time management needs, since the participants did not receive any incentives to participate. Also, this format is particularly advantageous for people living in remote areas or those with physical conditions that make travel to clinical settings challenging (29).

## 2 Material and methods

### 2.1 Participants and design

Thirteen adults (10 females and 3 males; M_age_ = 39.04, SD_age_ =13.74) provided informed consent for study participation. Their age varied between 18 and 58 years. Eleven participants had attained tertiary education, while two had completed high school. Urban residency was reported by twelve participants. The inclusion criteria were as follows: adults with T1DM diagnosed using a standard diagnostic system, who received their diagnosis for at least one year and were on a stable medication regimen throughout the entire study period. Also, we included in the trial patients with digital skills and access to the necessary technology used to provide the intervention. Exclusion criteria involved diagnoses of cognitive impairment, language impairment, severe psychiatric conditions (e.g., psychosis) or other conditions that could alter the participant’s capacity for understand and participate in the intervention (e.g., substance abuse). Additionally, patients with life-threatening conditions (e.g., multiple sclerosis) or chronic medical comorbidities were also excluded because medical complexities could overshadow the impact of the intervention on diabetes management. People participating in psychological counseling during the data collection period were not included in this study to minimize confounding effects.

To observe patients’ responses to an ACT intervention, this study used a single-arm trial design and the following outcomes were quantitatively measured before and after the intervention: stress, diabetes acceptance, PDR, and psychological flexibility. Additionally, using a qualitative approach, participants were asked for feedback after the intervention.

### 2.2 Measures

#### 2.2.1 Stress

The Depression Anxiety Stress Scale (DASS-21R) (30, 31) was used to assess patient stress. It is a self-report scale that assesses the negative emotional states of depression, anxiety, and stress on a 4-point Likert scale ranging from 0 to 3. The DASS-21R was validated on the Romanian population (28) and is divided into three subscales: one for each negative emotional state and one for overall distress. We used seven items of the stress subscale. An example of an item is ,,I found it difficult to relax”. The DASS-21R may be used in clinical settings to help with diagnosis and outcome tracking as well as in non-clinical contexts as a mental health screener (32). It is a dimensional instrument, and ratings highlight the degree to which someone experiences symptoms (30). The stress score was obtained by summing up the answers to the corresponding items. Bigger scores indicated higher levels of stress.

#### 2.2.2 Diabetes acceptance

The Acceptance and Action Diabetes Questionnaire (AADQ) (15) was used to assess this variable. The AADQ consists of six items and asks respondents to use a 5-point Likert scale (1 = “Never” to 5 = “Always”) to assess the degree to which they exhibit diabetes non-acceptance behaviors (for example, “I try to avoid reminders of my diabetes.”, “When I have an upsetting feeling or thought about my diabetes, I try to get rid of that feeling or thought.”). Smaller scores indicated a higher level of acceptance. For this study, the AADQ was translated from English to Romanian using forward and backward translation procedures.

#### 2.2.3 The patient-doctor relationship

This variable was assessed using the Patient Doctor Relationship Questionnaire (PDRQ-9) (33), a 9-item scale that includes questions about helpfulness (e.g., ‘‘My doctor helps me’’), trustworthiness (e.g., ‘‘I trust my doctor’’), understanding (e.g., ‘‘My doctor understands me’’), dedication, and accessibility of the doctor. The responses were graded on a 5-point Likert scale, with 0 being “not at all appropriate” and 4 being “totally appropriate”. Higher scores reflected better PDR. The scale was back-translated from English to Romanian.

#### 2.2.4 Psychological flexibility

The Multidimensional Psychological Flexibility Inventory (MPFI) (34) was used to examine psychological flexibility. It is a 60-item assessment that assesses ACT processes. Responses were graded on a 6-point Likert scale, with 1 being “never true” and 6 being “always true.” The measure provides two types of scores: global psychological flexibility and inflexibility (35). An example of an item is “I was receptive to observing unpleasant thoughts and feelings without interfering with them.”. The items for each of these subscales were summed up separately. Higher scores indicated higher levels of flexibility and inflexibility. The MPFI has been demonstrated to have strong convergent and discriminant validity as well as high internal consistency (α = .96) (34). The measure was back-translated into Romanian.

#### 2.2.5 Verbal feedback

At the end of the final session, participants were invited to give feedback on their experience with the ACT intervention. Specifically, they were asked: “How was it for you to take part in this intervention?” and “What did you like and what did you dislike about our sessions?”. The responses provided by the participants were recorded by the psychologist overseeing the sessions.

### 2.3 Intervention

The participants diagnosed with T1DM engaged in a series of four individual online ACT sessions conducted via the Zoom platform. This platform is widely used in Romania since the COVID-19 pandemic and also meets minimum security and privacy standards. These sessions, each lasting 45 minutes, were facilitated by a clinical psychologist who had no prior involvement in the participants’ clinical care preceding the trial. The intervention protocol was devised collaboratively by three researchers and clinical psychologists with expertise in the implementation of ACT.

The ACT sessions included diverse components, incorporating psychoeducation, mindfulness practices, defusion techniques, exploration of meaning, and goal-oriented value-setting strategies. *In the first session*, the ACT model was introduced, underscoring the study’s significance for individuals with diabetes and discussing the role of psychological flexibility in diabetes management. We started with mindfulness exercises (36) to ground the patients in the first session, and then psychoeducation about the importance of dropping an anchor (37) in moments of stress and anxiety. *The second session* delved into patient cognitions and emotions, emphasizing the cultivation of acceptance and emotional distance through defusion metaphors (38) such as “the clouds in the sky” or “the leaves on the lake”. The session finished with a mindfulness activity (i.e., mindfulness of sound or mindfulness of breath).

*The third session* centered on the exploration of patients’ values and life objectives, introducing the choice point model (39) Participants identified behaviors aligning with their values and those conflicting with them (40). The session encouraged participants to pause during internal struggles, observe, and record their experiences. Additionally, participants were prompted to select actions in harmony with their values and acknowledge behaviors misaligned with those values (39) The discussion extended to the significance of language in shaping self-perception and utilizing language as a defusion strategy (e.g., distinguishing between “I have the thought that I am sad” and “I am sad”) (41) *The last session* addressed the quest for life balance through metaphors and imagery related to health-promoting behaviors such as mindful eating and walking. The intervention concluded with verbal feedback from the participants.

### 2.4 Procedure

Participants were recruited through online Romanian diabetes groups and social media platforms from December 2022 to August 2023. They completed the baseline questionnaires online and participated in four individual ACT sessions. They were retested after the fourth session, the next day, using the same measures and asking them to provide verbal feedback regarding their experience with the intervention.

Prior to participation, the patients gave their consent, both verbally and in a written manner. They had the possibility of inquiring more information about the study and retained the autonomy to withdraw at any time without consequences. Participation was voluntary, no incentives were offered, and the intervention was free of charge. Approval for the research protocol was secured from the Ethic Committee of the County Emergency Clinical Hospital ,Sf. Apostol Andrei”, Galati (notice no. 16275, 27.07.2023).

### 2.5 Statistical analyses

Statistical analyses were conducted using IBM SPSS Statistics, version 25. Initial reporting involved the computation of basic descriptive statistics (means, standard deviations, normality indicators of the distributions). Correlations were not included due to the risk of inflating the link, as a result of the small sample size (42). To estimate the normality of the distribution, we used the interval [-1.96; + 1.96] as the cutoff (43). Given the small sample size, to assess the participants’ to the individual intervention, pre- and post-intervention differences for each measured variable were computed with the non-parametric Wilcoxon signed-rank test (44), with a significance threshold set at α = .05. Post-hoc power calculations were employed using G*Power (version 3.1), to assess the sensitivity of the study in detecting the observed outcomes.

## 3 Results

### 3.1 Feasibility

A total of 47 adults initially completed the behavioral measures and expressed an intention to participate. However, 10 individuals failed to attend the first session, citing reasons such as time constraints or difficulties in installing the Zoom platform. Efforts to re-engage these individuals, even as controls, were unsuccessful. Context-specific factors may be led to this outcome: psychological interventions face considerable stigma (45). This cultural backdrop contributes to reluctance among potential participants, particularly when such services are not explicitly recommended by their physician. Our clinical experience, consistent with observations in the broader Romanian healthcare context, indicates that people with lower educational levels exhibit less propensity towards participating in psychological interventions. Unfortunately, a lack of comprehensive, official reports on these phenomena within Romania limits our ability to quantify this effect precisely.

**Figure 1:**
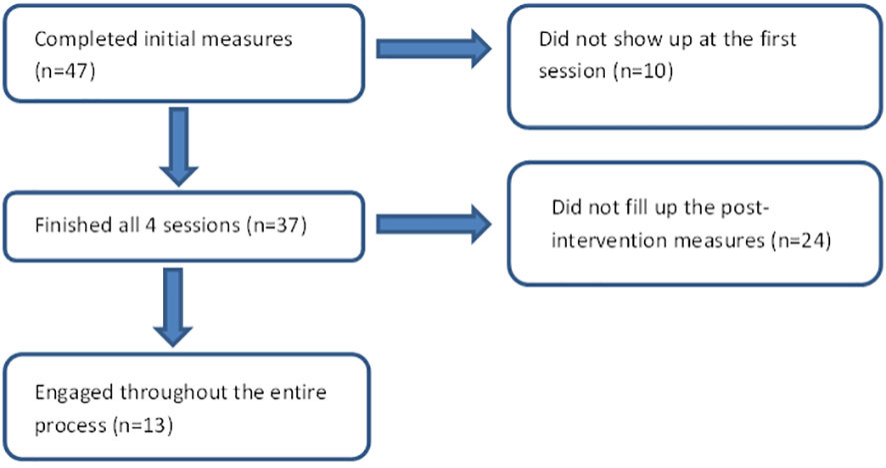
Study flowchart.

Twenty-four participants engaged in the four online sessions but did not complete the post-intervention measures, primarily attributed to time constraints, especially during the summer holidays, or unavailability of an internet connection. Despite researchers’ attempt to help these participants fill in the post-intervention measures, they did not follow through. However, verbal feedback was obtained from them at the last ACT session. As such, only 13 participants were fully engaged in the entire process (see **Figure 1**). No adverse events or technical issues were present during any of the sessions.

### 3.2 Descriptive statistics

**Table 1** shows the main descriptive indicators of the variables (mean, standard deviation, skewness, and kurtosis). Among all measured variables, the post-test distribution of psychological inflexibility showed significant deviations from the condition of normal data distribution.

**Table 1:** Descriptive statistics of measured variables.

Variable	Pre intervention				Post intervention			
	Mean	SD	Skewness	Kurtosis	Mean	SD	Skewness	Kurtosis
Stress	30.46	9.80	-.18	-1.4	11.85	9.43	.57	-.52
Acceptance	17.77	5.1	-.34	-1.01	8.54	3.12	1.28	1.18
PDR	32.92	7.92	-.23	-.83	35.08	10.91	-1.45	1.78
Flexibility	3.08	.59	-.87	-.05	4.8	.93	-.5	-1.01
Inflexibility	3.58	.82	.01	-1.14	2.70	.84	1.21	2.11

### 3.3 Outcome measures

As displayed in **Table 2**, there is a statistically significant difference between the diabetes associated stress (Z = 86, p = .005, r = .89), diabetes acceptance (Z = 78, p = .002, r = .99), psychological flexibility (Z = 3.50, p = .004, r = -.92), and psychological inflexibility (Z = 80, p = .01, r = .75) before and after the intervention, with large effect sizes. In other words, stress levels and inflexibility diminished, while flexibility and diabetes acceptance increased, as anticipated. However, for PDR, the results did not change significantly.

**Table 2:** Pre- and post-intervention differences.

Variables	Wilcoxon test Z	p	Pre- and post-intervention mean differences	Effect size (r)
Stress	86	.005	18	.89
Acceptance	78	.002	10	.99
PDR	13.50	.168	-5	-.50
Flexibility	3.50	.004	-1.70	-.92
Inflexibility	80	.013	.86	.75

The post-hoc power analysis conducted using G*Power was based on the observed effect sizes (from.50 to.99), derived from the Wilcoxon test, with a total sample size of 13 participants, an alpha level set at 0.05, two tailed, and minimum asymptotic relative efficiency parent distribution. For PDR, the analysis indicated an achieved power of.33, suggesting that the study was underpowered for detecting smaller effects of the intervention. However, for the other variables, the post-hoc power analysis indicated an achieved power between.72 and.84, around the conventional threshold of.80 (46). These findings underscore the necessity for larger-scale studies to further investigate the potential benefits of the intervention with increased statistical sensitivity.

### 3.4 Verbal feedback

At the end of all four sessions, the participants had the opportunity to articulate their impressions regarding the intervention. All the participants that finished the four sessions (N = 37) reported they were satisfied with the outcomes. Participant A., a 25 year-old female, expressed a sense of connection with her body after dealing with experiential avoidance for many years: “*I feel more connected with my body after talking to you*”. She said *,,My initial belief was that by avoiding and denying emotions, I would finally get rid of all the stress generated by T1DM*”. Another participant, identified as B., a 52 year-old female with over two decades of experience managing T1DM, acknowledged her ongoing struggle to align with her values and derive motivation for adherence to exercise and dietary regimens. She said: “*It is so easy and at the same time hard to talk about values, that I have never thought about what is important for me and how to use my dear ones as a motivation to deal with diabetes.*”

## 4 Discussion

This study aimed to assess the impact of a four-week online individual ACT intervention in T1DM patients from Romania. Quantitative results indicated a statistically significant expected difference between diabetes-associated stress, diabetes acceptance, and psychological flexibility before and after the intervention. However, PDR did not change significantly. Qualitative feedback from participants conveyed a positive evaluation of the intervention.

Overall, the results align with previous findings, suggesting that ACT interventions help patients with diabetes lower their stress levels and improve their acceptance, psychological flexibility, and depression (15, 17, 47, 48). The positive outcomes obtained in such a short time are, most likely, due to the transdiagnostic and multicomponent nature of the intervention. Also, the ACT intervention was delivered individually, which allowed the personalization of therapeutic activities according to the situation of each participant. The psychoeducation stage helped the patients to consolidate their knowledge about diabetes management and normalize their attitude towards it, a process that has therapeutic effects. Furthermore, it familiarized them with the hexaflex model and offered predictability regarding their therapeutic activities. The benefits of psychoeducation for patients with diabetes have already been reported in the literature (49).

With the help of mindfulness techniques, participants learned how to reduce the physiological component of stress, a well-documented effect of these (50). The cognitive component is addressed through cognitive defusion techniques that change the function of the thought without necessarily modifying its content (8). These techniques normalize attitudes towards negative repetitive mental processes and facilitate thought acceptance. Ultimately, the participants learned how to find meaning in their lives and value-driven goal setting despite the presence of diabetes in their lives. Studies have shown that finding meaning in life reduces repetitive negative thinking (51) and it is a factor in protection and adaptation to daily stressors (52) Greater adaptation skills result in greater flexibility, which is essential for good health (53) Identifying personal values and using them to guide patients’ actions addressed the behavioral component of change, and it should also enhance meaning in life, as opposed to simple goal-setting training (54). The qualitative feedback received from the participants supported the explanations provided by scientific data. Participants referred to how helpful it was to talk about their values and accept the emotions and thoughts associated with illness without being asked specific questions in this direction.

Concerning PDR, our results did not show a significant difference between the two measurement points. This could be due to several reasons: the lack of PDR focused intervention, the small number of participants in the current study, and the complexity of variables involved in this kind of relationship. ACT is a transdiagnostic intervention and the therapeutic activities did not focus on the PDR, but rather on diabetes-related content, which may be insufficient to capture changes in the PDR. A PDR-targeted ACT approach could specifically focus on fostering communication skills, mutual understanding, and empathy, thereby facilitating a more collaborative and effective management plan. This could be particularly beneficial in contexts where chronic disease management requires ongoing patient-provider interaction and adjustment to treatment plans. The current sample size was too small to capture changes. Moreover, trust in medical doctors is a dynamic construct that varies based on sociocultural context. For example, a cross-sectional survey from India (55) conducted on a sample of 625 men and women from urban and rural areas concluded that trust in medical doctors was largely influenced by subjective factors (e.g., respect for the doctor, predictability in receiving treatment) rather than objective factors (e.g., doctor’s competence). The behavior/communication skills of medical doctors and their level of comfort seemed to impact the degree of trust (7). Therefore, future studies should investigate PDR from the perspective of culture-specific factors, empathy, mutual understanding, and psychological flexibility.

These preliminary results encourage the use of ACT for the management of patients with diabetes. Even so, it is necessary to consider them in light of the limitations of this study. First, this study did not include a control group, which limits the methodological robustness of the results. Second, the sample size was small and less than 50% of the participants who initially showed interest were able to attend the sessions and complete the questionnaires. Therefore, the results are prone to “survivor bias.” One explanation may be the lack of material incentives. Another explanation may be that the diabetologist did not refer the patients for the study. In addition, the participants were young and mature adults, and their digital skills may not have been well-developed, even if they reported so before the study. It is estimated that approximately 30% of Romanians have basic digital skills (56). Some participants had difficulty installing the application on which the sessions were held. Another explanation may be related to the limited time patients have at their disposal and difficulty in managing all tasks in their lives (57) However, we believe that the individual online format was helpful in this regard. If the use of psychological services would be an integral part of the diabetes management process, patients will likely be more interested in participating. Even so, this is not the case in Romania, which means that patients and medical doctors should be educated and trained in multidisciplinary practice.

Given the limitations and challenges associated with this pilot study, the following changes can be considered for the full trial, to improve retainment and intervention outcomes: to offer pre-trial training sessions on digital tools used in the study, to contact medical staff throughout the country and ask them to refer patients for the trial, to include targeted activities aimed at improving the patient-doctor relationship, to provide incentives for participants completing the intervention. Additionally, the methodology could be improved by adding a control group and a follow-up measurement at one month after the intervention.

## 5 Conclusions

The current data showed that the brief online ACT intervention had a positive impact on patients with diabetes, diminishing stress levels, increasing acceptance of diabetes, and psychological flexibility. The results are in line with research conducted in other countries and reinforce the necessity of a multidisciplinary approach in diabetes management. PDR did no exhibit significant changes, potentially due to the study’s focus on diabetes-related content rather than targeted PDR intervention. Qualitative feedback reinforced the positive impact of the ACT intervention. Despite the study’s limitations, the data is encouraging and provides evidence to invest in a larger trial.

## Data availability statement

The raw data supporting the conclusions of this article will be made available by the authors, without undue reservation.

## Ethics statement

The studies involving humans were approved by Ethic Committee of the County Emergency Clinical Hospital ,,Sf. Apostol Andrei”, Galati (notice no. 16275, 27.07.2023). The studies were conducted in accordance with the local legislation and institutional requirements. The participants provided their written informed consent to participate in this study.

## Author contributions

CS: Conceptualization, Investigation, Methodology, Project administration, Writing – original draft, Writing – review & editing. AT: Conceptualization, Supervision, Writing – review & editing, Project administration. AN: Conceptualization, Project administration, Supervision, Writing – review & editing. CI: Data curation, Formal analysis, Writing – original draft, Writing – review & editing. ES: Investigation, Writing – original draft. SN: Investigation, Writing – original draft. GH: Formal analysis, Writing – original draft.
